# Identification of Active Denitrifiers in Rice Paddy Soil by DNA- and RNA-Based Analyses

**DOI:** 10.1264/jsme2.ME12076

**Published:** 2012-09-05

**Authors:** Megumi Yoshida, Satoshi Ishii, Daichi Fujii, Shigeto Otsuka, Keishi Senoo

**Affiliations:** 1 Department of Applied Biological Chemistry, The University of Tokyo, 1–1–1 Yayoi, Bunkyo-ku, Tokyo 113–8657, Japan

**Keywords:** denitrification, *nirS*, *nirK*, *nosZ*

## Abstract

Denitrification occurs markedly in rice paddy fields; however, few microbes that are actively involved in denitrification in these environments have been identified. In this study, we used a laboratory soil microcosm system in which denitrification activity was enhanced. DNA and RNA were extracted from soil at six time points after enhancing denitrification activity, and quantitative PCR and clone library analyses were performed targeting the 16S rRNA gene and denitrification functional genes (*nirS*, *nirK* and *nosZ*) to clarify which microbes are actively involved in denitrification in rice paddy soil. Based on the quantitative PCR results, transcription levels of the functional genes agreed with the denitrification activity, although gene abundance did not change at the DNA level. Diverse denitrifiers were detected in clone library analysis, but comparative analysis suggested that only some of the putative denitrifiers, especially those belonging to the orders *Neisseriales*, *Rhodocyclales* and *Burkholderiales*, were actively involved in denitrification in rice paddy soil.

Denitrification is a microbial reaction in which NO_3_^−^ and NO_2_^−^ are successively reduced to NO, N_2_O and N_2_ (16, 47). It is an important part of the nitrogen cycle in the biosphere (8, 28). In upland agricultural fields, denitrification can cause the loss of fertilizer nitrogen and the emission of N_2_O, a greenhouse gas. Although denitrification commonly occurs in rice paddy soil, the emission of N_2_O gas is much lower than in upland crop fields (1, 16, 25). This indicates that microbes are highly active in N_2_O reduction in rice paddy field soils.

Because many types of bacteria (3, 6, 20), archaea (8, 27) and fungi (8, 35) are known to have the ability to denitrify, it is difficult to detect denitrifiers by a culture-independent approach using the 16S rRNA gene alone (28). Instead, primers targeting the cytochrome *cd*_1_-containing nitrite reductase gene (*nirS*) and the copper-containing nitrite reductase gene (*nirK*) are frequently used as marker genes to examine denitrifier communities in various environmental samples (4, 17, 18, 29, 31, 38, 44). In addition, the nitrous oxide reductase gene (*nosZ*) can be used to detect N_2_O-reducing denitrifiers (10, 15, 22, 30).

Previously, we analyzed the community structure of the denitrifiers in rice paddy soil by DNA-based clone library analysis targeting *nirS* and *nirK* (45, 46). In these studies, clones related to the *nirS* of *Burkholderiales* and the *nirK* of *Rhizobiales* were frequently detected in soil with strong denitrification activity; however, clones distantly related to the known denitrifiers were also detected. Although DNA-based analysis is useful for examination of the structure of denitrifier communities, it is not clear whether these microbes actually perform denitrification. RNA-based analysis can detect the transcription of functional genes; therefore, it can be used to detect active denitrifiers in the environment (9, 21, 26, 34, 36, 43).

Consequently, the objectives of this study were (1) to analyze the amount of bacterial 16S rRNA and the transcription levels of *nirS* and *nirK*, (2) to analyze microbial community structures before and after inducing denitrification, (3) to examine the diversity of *nirS*, *nirK* and *nosZ* and their transcripts and (4) to compare results obtained by DNA- and RNA-based analyses.

## Materials and Methods

On 23 April 2009, soil was collected from a rice paddy field at Niigata Crop Research Center, Niigata Agricultural Research Institute, Nagaoka, Niigata, Japan (37°44′N, 138°87′E). The soil samples were sieved with 2-mm mesh to remove gravel and plant roots, and the samples were stored at 4°C. The soil type was gley soil. Physicochemical characteristics of the soil have been described elsewhere (12).

A previously established laboratory soil microcosm system (32) was used with modifications in this study. In brief, 5 g moist soil (corresponding to *ca.* 3 g air-dried soil) was pre-incubated in a serum vial at 30°C for 1 week with 5.4 mL sterilized distilled water to decrease soil redox potential. After pre-incubation, excess water was removed, and 0.3 mg N NO_3_^−^ and 1.5 mg C succinate were added as an electron acceptor and electron donor, respectively, for denitrification. The vials were then anaerobically incubated with Ar gas at 30°C. Soil samples were collected from replicated vials (n=3) after 0-, 6-, 12-, 16-, 20- and 24-h incubation. Another set of vials was incubated with Ar-C_2_H_2_ (90:10, v/v) to measure denitrifying activities in the soil microcosm by the C_2_H_2_ block method (32).

DNA was extracted and purified from 0.5 g soil using an UltraClean Soil DNA Isolation kit (MoBio Laboratories, Carlsbad, CA, USA). Purified DNA was diluted 50- and 10-fold for PCR and quantitative PCR (qPCR), respectively, to adjust the DNA concentration to the proper condition for PCR, and to reduce the influence of the PCR inhibitors (*e.g.*, humic substances).

RNA extraction was performed as described previously (41). RNA was extracted from 3.2 g soil using a RNA PowerSoil Total RNA Isolation kit (MoBio Laboratories). Extracted RNA was purified using Illustra MicroSpin Columns S-400 HR (GE Healthcare, Buckinghamshire, UK) to remove humic substances, and the remaining DNA was removed using a Turbo DNA-free kit (Applied Biosystems, Foster City, CA, USA). RNA was further purified and concentrated using an RNA Clean & Concentrator-5 kit (Zymo Research, Orange, CA, USA). The amount of purified RNA was measured using a NanoDrop 1000 (NanoDrop Products, Wilmington, DE, USA). The absence of DNA carryovers in the RNA samples was verified by PCR targeting the 16S rRNA gene without reverse transcription. Purified RNA was reverse transcribed using random hexamers and PrimeScript Reverse Transcriptase (Takara Bio, Otsu, Shiga, Japan) with 20 U of SUPERase-In RNase Inhibitor (Applied Biosystems). Synthesized cDNA was diluted 10-fold for PCR and qPCR, and 1,000-fold for qPCR targeting 16S rRNA gene. Replicate DNA and cDNA samples were pooled and used for clone library analyses.

qPCR was performed using a StepOne Real-Time PCR System (Applied Biosystems) with Power SYBR Green PCR Master Mix (Applied Biosystems). Primers 357F and 520R (19, 24), modified cd3aF and R3cd (17), and nirK876F and nirK1040R (17) were used for quantification of the bacterial 16S rRNA gene, *nirS*, and *nirK*, respectively. PCR was performed under the conditions described elsewhere (5, 17, 23). Amplification of correctly sized products was verified by dissociation curve analysis and agarose gel electrophoresis. Statistical significance among the amount of genes and gene transcripts was examined by analysis of variance at α=0.05 using R program version 2.8.1, as previously described (13).

For clone library analysis, DNA and cDNA samples were amplified with the modified primers 27F and 1492R (39), Cd3aF and R3cd (38), F1aCu and R3Cu (38) and nosZ-F-1181 and nosZ-R-1880 (30) for the bacterial 16S rRNA gene, *nirS*, *nirK* and *nosZ*, respectively. These primers, different from those used for qPCR, were employed because longer PCR products were suitable for clone library analysis. The PCR reaction mixture (20 μL) contained 10 mM Tris-HCl (pH 7.5), 10 mM NaCl, 0.01 mM EDTA, 0.2 mM dithiothreitol, 5% (w v^−1^) glycerol, 1.5 mM MgCl_2_, 0.2 mM each dNTP, 1 μM each of the forward and reverse primers, 10 μg bovine serum albumin, 2 units of BioTaq HS DNA polymerase (Bioline, London, UK) and 1 μL soil DNA or cDNA. After incubation for 10 min at 95°C, PCR was performed in a thermal cycler, the GeneAmp PCR System 9700 (Applied Biosystems), under the conditions described elsewhere (30, 38, 39). For samples with low concentrations of PCR template (*nirK* and *nosZ* amplicons from RNA samples and *nirS* amplicons from RNA samples extracted from the soil before incubation), a second PCR reaction was performed using a 10-fold diluted product of the first PCR reaction as the template. The PCR products were ligated into the pGEM-T vector system (Promega, Madison, WI, USA) and transferred to *Escherichia coli* JM109 high-efficiency competent cells (Promega) according to the manufacturer’s instructions. Colonies were randomly selected, and the inserted fragments were sequenced as described previously (45).

The 16S rRNA clones sharing nucleotide sequence homology at more than 99% were grouped into one operational taxonomic unit (OTU) using DOTUR program ver. 1.53 (33). The 16S rRNA clones were classified into bacterial taxa using the Ribosomal Database Project classifier program (40). Sequences from *nirS*, *nirK* and *nosZ* clones were translated, and OTU values and diversity indices were calculated as described previously (46). Phylogenetic trees were constructed using ClustalX ver. 1.83 based on the nucleotide sequences of 16S rRNA or 16S rRNA gene, and the deduced amino acid sequences from *nirS*, *nirK* and *nosZ*.

The nucleotide sequences obtained in this study have been deposited in the DDBJ, EMBL and GenBank databases under the following accession numbers: AB672106–AB672500.

## Results

**Fig. 1:**
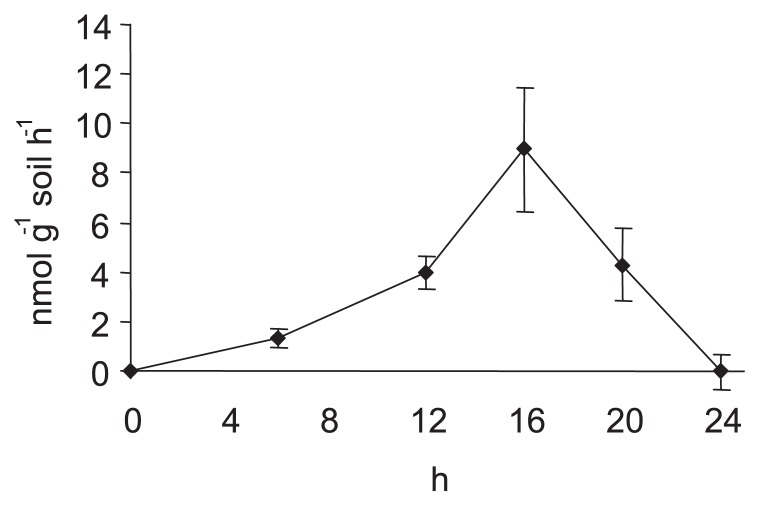
Denitrifying activities in the soil microcosm system as measured by the C_2_H_2_ block method.

The denitrification activity based on N_2_O production increased with incubation time and peaked 16 h after the start of incubation (Fig. 1).

**Fig. 2:**
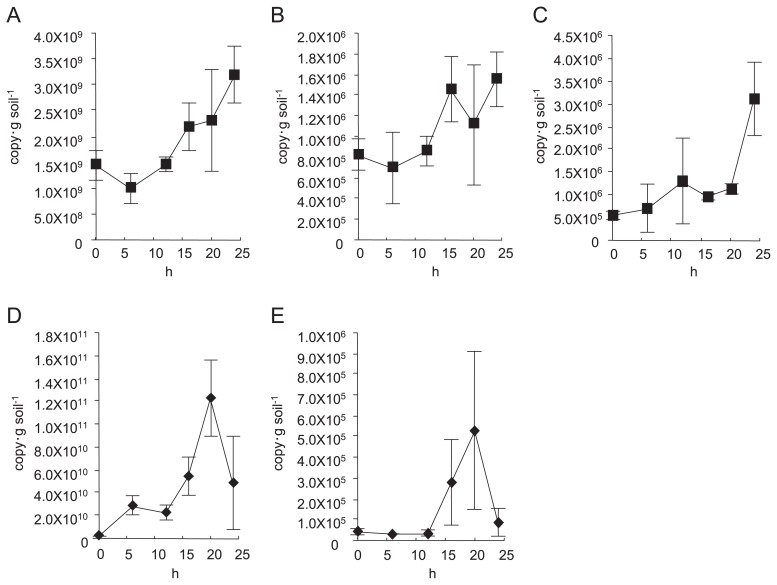
Changes in the amounts of (A) 16S rRNA gene, (B) *nirS*, (C) *nirK* from DNA samples and (D) 16S rRNA, (E) *nirS* transcripts from cDNA samples. X axes show incubation time. Y axes show numbers of gene copies or gene transcripts.

Based on DNA-based analysis, copy numbers of the 16S rRNA gene and *nirK* increased as the incubation proceeded (*p*=0.014 and *p*=0.006) (Fig. 2A and C), whereas the amount of *nirS* did not show a significant change (Fig. 2B). Conversely, based on RNA-based analysis, the amount of 16S rRNA significantly increased and peaked 20 h after the start of incubation (*p*=0.002). The amount of *nirS* showed a similar trend to that of 16S rRNA, but the tendency was not significant (*p*=0.13) (Fig. 2E). The amount of *nirK* product was below the detection limit.

Clone library analysis was performed with DNA and RNA samples obtained from soil before incubation and after 20-h incubation, the time-point at which the amount of 16S rRNA transcripts peaked (Fig. 2D). The numbers of obtained clones are shown in Table 1. We could not obtain *nosZ* amplicons from the RNA samples extracted from the soil before incubation. All of the *nirK* clones obtained from the RNA samples extracted from the soil before incubation and half of the *nirK clones* obtained from the soil after incubation were pseudogenes, and therefore, they were removed from subsequent analysis.

**Fig. 3:**
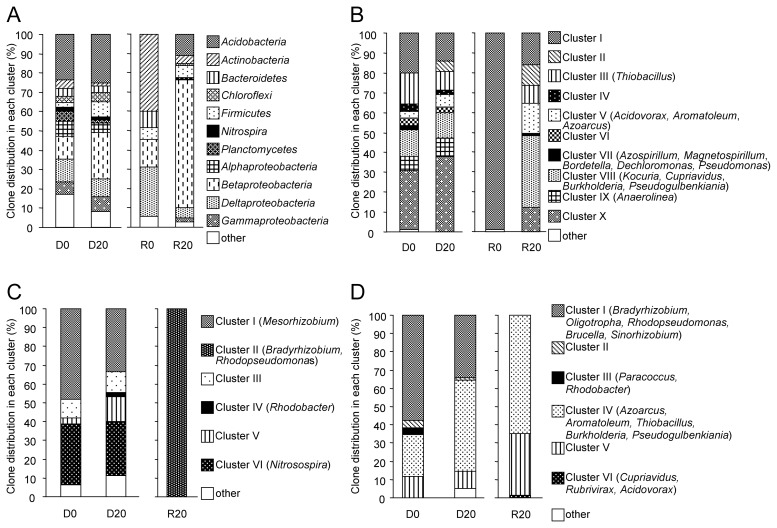
Changes in the distribution of (A) 16S rRNA, (B) *nirS*, (C) *nirK* and (D) *nosZ* clones in response to denitrification-inducing conditions. Clones were grouped into clusters based on the phylogenetic trees shown in Supplementary Materials (Fig. S1, 2, 3, and 4).

Clones of the 16S rRNA gene were widespread in the phylogenetic tree (Fig. S1). Based on DNA-based analysis, community structures were similar between soil samples before and 20 h after incubation (Fig. 3A); however, RNA-based analysis showed a marked population change between these samples (Fig. 3A). In addition, the diversity in the 16S rRNA gene was larger in the DNA samples than in the RNA samples (Table 1). In the RNA samples, proportions of the clones related to the class *Betaproteobacteria* and the phylum *Acidobacteria* increased by 20-h incubation from 14.3% to 66.2% and from 0.0% to 10.8%, respectively. Within *Betaproteobacteria*, clones related to the orders *Neisseriales* and *Rhodocyclales* accounted for a large proportion. In contrast, the proportions of clones related to the phyla *Actinobacteria* and *Bacteroidetes* and the class *Deltaproteobacteria* were decreased by incubation from 40.0% to 4.1%, 8.6% to 0% and 25.7% to 5.4%, respectively.

Clones of *nirS* were also widespread in the phylogenetic tree (Fig. S2). Similar to the results based on the 16S rRNA gene, the composition of clones did not change significantly in the DNA samples, whereas that of clones in the RNA samples changed greatly. In addition, the diversity of *nirS* was larger in the DNA samples than in the RNA samples (Table 1). In the RNA samples, almost all *nirS* clones obtained from soil before incubation were grouped in Cluster I, whereas clones from soil after incubation were spread throughout the phylogenetic tree. Clones obtained from both DNA and RNA samples were found in Clusters I, II, III, V, VII, VIII, and X. Although clones in Clusters I, II, and X were distantly related to the *nirS* of known denitrifiers, those in Cluster III were related to the *nirS* of *Hydrogenophilales* bacteria (*Thiobacillus* spp.), those in Cluster V to the *nirS* of *Rhodocyclales* bacteria (*Acidovorax* spp. and *Aromatoleum* spp.), those in Cluster VII to the *nirS* of *Rhodospirillales* bacteria (*Azospirillum* spp. and *Magnetospirillum* spp.), *Burkholderiales* bacteria (*Bordetella* spp.), *Rhodocyclales* bacteria (*Dechloromonas* spp.) and *Pseudomonadales* bacteria (*Pseudomonas* spp.), and those in Cluster VIII to the *nirS* of *Burkholderiales* bacteria (*Burkholderia* spp. and *Cupriavidus* spp.) and *Actinobacteria* (*Kocuria* spp.). Clones in Cluster VIII accounted for 36.4% of clones obtained from the RNA samples after incubation (Fig. 3B). All clones in Clusters IV, VI and IX were from DNA samples, not from RNA samples.

The *nirK* clones were also widespread in the phylogenetic tree (Fig. S3); however, the sequence compositions were different between DNA and RNA samples. The diversity indices of *nirK* were smaller than those of the other functional genes (Table 1). All clones found in Clusters I, III, IV, V, and VI were obtained only from DNA samples. On the other hand, clones in Clusters II were obtained only from RNA samples (Fig. 3C).

Clones of *nosZ* were also widespread in the phylogenetic tree (Fig. S4). Clones in Cluster I were related to the *nosZ* of *Rhizobiales* (*Bradyrhizobium* spp., *Brucella* spp. and *Sinorhizobium* spp.) and were only observed in the DNA samples. The proportion of the clones in Cluster I decreased after 20-h incubation (Fig. 3D), suggesting that these clones were not actively involved in N_2_O reduction. In contrast, the proportion of clones in Cluster IV that increased after incubation and were dominant in the RNA samples (related to *Azoarcus* spp., *Aromatoleum* spp., *Thiobacillus* spp., *Burkholderia* spp. and, *Pseudogulbenkiania* spp.) and Cluster V (distantly related to known bacteria) were also dominant in cDNA.

## Discussion

In this study, denitrifier communities were investigated both by DNA- and RNA-based analyses targeting 16S rRNA and denitrification functional genes, and their transcripts. DNA-based analysis revealed that the denitrifier community did not change much by incubation, whereas RNA-based analysis showed dynamic changes in the abundance and composition of transcripts as a result of incubation (Fig. 3). The results of this study were compared with those obtained previously by culture-dependent analyses (14, 37) and DNA-based culture-independent analyses (45, 46) with rice paddy field soils. In the microcosm setup used in this study, the amount of 16S rRNA and *nirS* transcripts increased along with denitrification activity. In contrast to the RNA-based study, the amount of each gene from DNA samples did not change significantly within 24-h incubation. In addition, the amounts of the functional gene transcripts (mRNA) were markedly smaller than those of the copy numbers (DNA). These results indicated that only a few microbes may have transcribed their denitrification functional genes, and their growth was not significant enough to contribute to the overall increase in the amount of the functional genes within 24-h incubation; however, degradation of mRNA may also have caused the relatively small number of gene transcripts. Härtig reported the half-life of *nirS* transcripts to be 12.6 min (7), indicating that mRNA may be degraded during extraction.

Active microbes under denitrification-inducing conditions were identified by comparative analysis of the clone libraries. Although diverse bacteria were present in the rice paddy soil, as observed in a previous study (11), only a few were active under denitrification-inducing conditions. Among these, many clones related to the class *Betaproteobacteria*, especially those of the order *Neisseriales*, were the most abundant. Previous culture-based analysis also identified many *Neisseriales*-related bacteria (*e.g. Pseudogulbenkiania* sp. NH8B) in the same rice paddy soil (37). In addition to *Betaproteobacteria*, the proportion of clones related to the phylum *Acidobacteria* also increased after 20-h incubation. These microbes may also be involved in denitrification or N_2_O reduction. Based on genome analysis, some *Acidobacteria* may have the ability to reduce nitrate and nitrite (42). On the other hand, the proportion of 16S rRNA related to the phyla *Actinobacteria* and *Bacteroidetes* and the class *Deltaproteobacteria* decreased. These microbes may be present in soil, but they did not respond to denitrification-inducing conditions.

Similar to comparative clone library analysis based on the 16S rRNA or the 16S rRNA gene, analysis of the denitrification functional gene or the gene transcripts suggested that only a small number of denitrifiers were active under denitrification-inducing conditions. By comparative analysis of the clone libraries, we were able to identify the *nirS* and *nosZ* clones that increased in response to denitrification-inducing conditions. Some of these were distantly related to the *nirS* of known denitrifiers, suggesting that denitrifiers carrying these functional gene sequences have not been isolated yet. In Cluster X, the *nirS* clone sequences obtained in this study from the rice paddy soil in Niigata were similar to the *nirS* from *Bradyrhizobium* sp. TSA1, which was isolated previously from rice paddy soil in Tokyo (14). Similar *nirS* sequences were also obtained from clone library analyses based on rice paddy soil in Tokyo (45, 46), indicating that these denitrifiers may be present in various types of rice paddy soils. Many clones related to the *nirS* of *Actinobacteria* and *Betaproteobacteria* (Cluster VIII in Fig. S2) and the *nosZ* of *Betaproteobacteria* (Cluster IV in Fig. S4) were frequently obtained from the soil when denitrification was actively performed, indicating that these bacteria may also play important roles in denitrification and N_2_O reduction.

In contrast to *nirS* transcripts, the number of *nirK* transcripts was not detected. This indicated that most of the active denitrifiers in this soil may use NirS, not NirK, for nitrite reduction. A previous culture-based study also reported that most denitrifiers isolated from rice paddy soil carried *nirS* (14); however, bias caused by the primers used in this study may also have caused the constant level of *nirK* expression. Although the contribution of *nirK*-carrying denitrifiers may not be large, we identified *nirK* clone sequences that were related to clone sequences of the order *Rhizobiales* (Cluster II) in RNA samples obtained from the soil after incubation. These microbes may have been involved in denitrification in the soil used in this study. Similar to the present study, these denitrifiers were previously isolated from rice paddy soils (2, 14, 37).

In conclusion, denitrifiers belonging to the class *Betaproteobacteria* (*e.g.* the orders *Neisseriales*, *Rhodocyclales* and *Burkholderiales*) may be involved in denitrification in rice paddy soils under the conditions used. In addition, denitrifiers harboring previously uncharacterized *nirS* may be involved in denitrification. Isolation and characterization of the denitrifiers carrying these genes (*e.g.*, 14) are necessary in the future. Furthermore, the soil was incubated with succinate in this study, however, it was reported that the denitrifier community responded differently to the carbon source (9). Further studies using diverse carbon sources may be needed for greater understanding of the active dentrifying community. Our study also clearly showed that only some of the denitrifiers actively performed denitrification in the environment. Although DNA-based analysis is useful to assess the diversity of denitrifier populations, an RNA-based approach is important for the identification of active denitrifiers.

## Supplementary Material



## Figures and Tables

**Table 1:** Diversity indices of the clone libraries obtained in this study

Name of soil sample		clones	OTUs	ChaoI	Shannon (*H′*)	Simpson (1/D)
D0	16S rRNA	93	82	637.0	4.342	267.375
	NirS	84	43	64.2	3.499	34.515
	NirK	31	7	8.0	1.410	3.370
	NosZ	26	12	19.5	2.302	12.500
D20	16S rRNA	63	54	348.0	3.901	130.200
	NirS	129	51	76.3	3.676	42.557
	NirK	45	7	8.0	1.648	4.853
	NosZ	82	17	24.0	2.199	5.796
R0	16S rRNA	35	19	26.5	2.781	22.037
	NirS	89	9	12.3	0.811	1.514
R20	16S rRNA	148	63	149.0	3.455	16.383
	NirS	99	21	26.3	2.527	8.601
	NirK	23	2	2.0	0.669	1.992
	NosZ	88	8	8.5	1.496	3.455

D0, DNA extracted from the soil before incubation; D20, DNA extracted from the soil after incubation; R0, cDNA extracted from the soil before incubation; R20, cDNA extracted from the soil after incubation.
