# Supplementary material for: Identification of Active Denitrifiers in Rice Paddy Soil by DNA- and RNA-Based Analyses
Source: Microbes Environ. 2012 Sep 5;27(4):456–61. doi: 10.1264/jsme2.ME12076 (PMC4103554; doi:10.1264/jsme2.ME12076)
Supplement: Supplementary file 1 [file 27_456_s1.pdf]

Supplementary data

Identification of Active Denitrifiers in Rice Paddy Soil by DNA- and RNA-Based Analyses

Megumi Yoshida[1], Satoshi Ishii[1,†], Daichi Fujii[1], Shigeto Otsuka[1] and Keishi Senoo[1]

[1]Department of Applied Biological Chemistry, The University of Tokyo, 1-1-1 Yayoi, Bunkyo-ku, Tokyo 113-8657, Japan

[†]Present address: Division of Environmental Engineering, Hokkaido University, Kita 13, Nishi 8, Sapporo, Hokkaido 060-8628, Japan

Email: myoshida_a@yahoo.co.jp

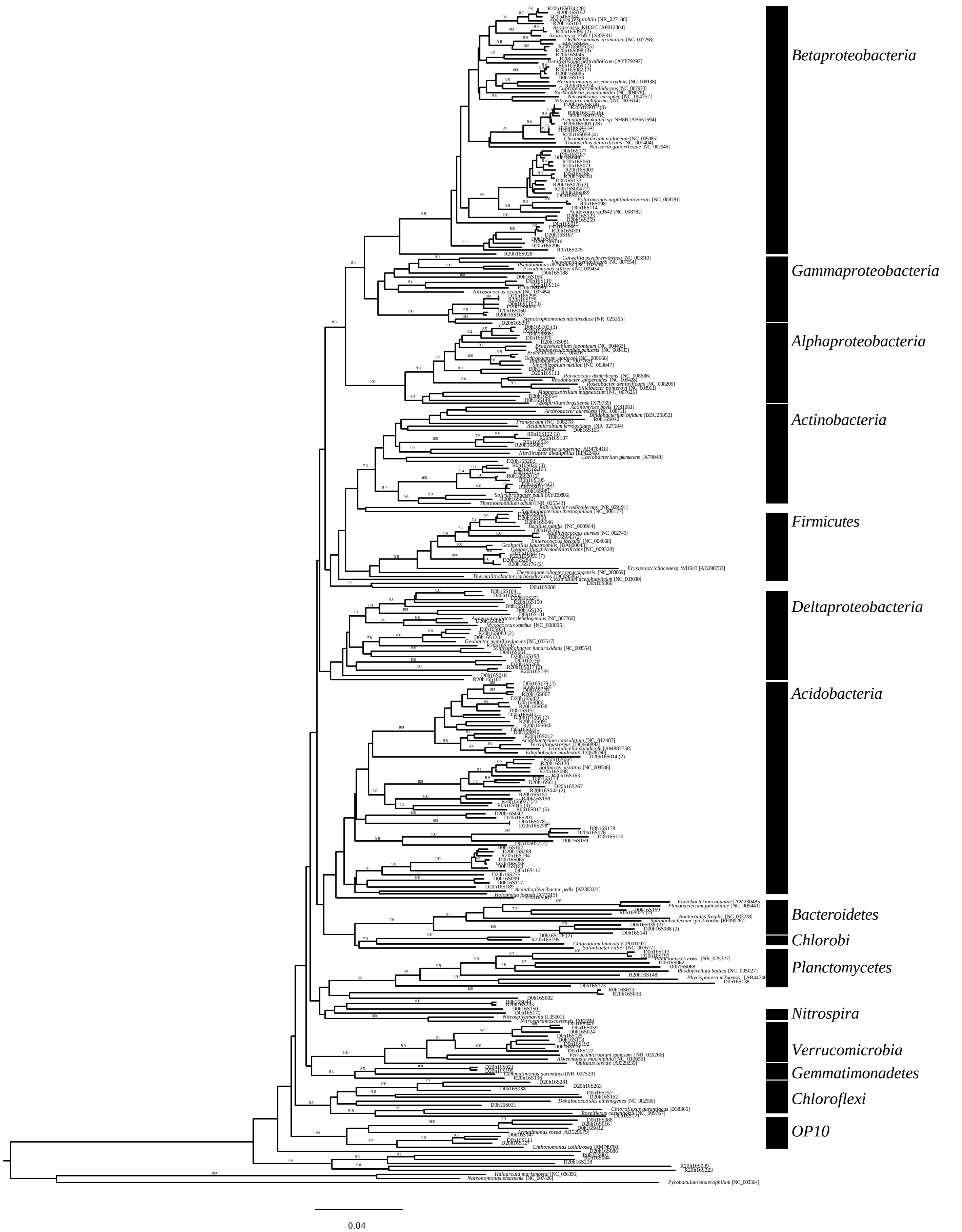

Figure S1   Neighbor-joining tree based on the sequences of 16S rRNA clones. Numbers in parenthesis represent the total number of clones in the OTU. Scale bar represents substitutions per site.

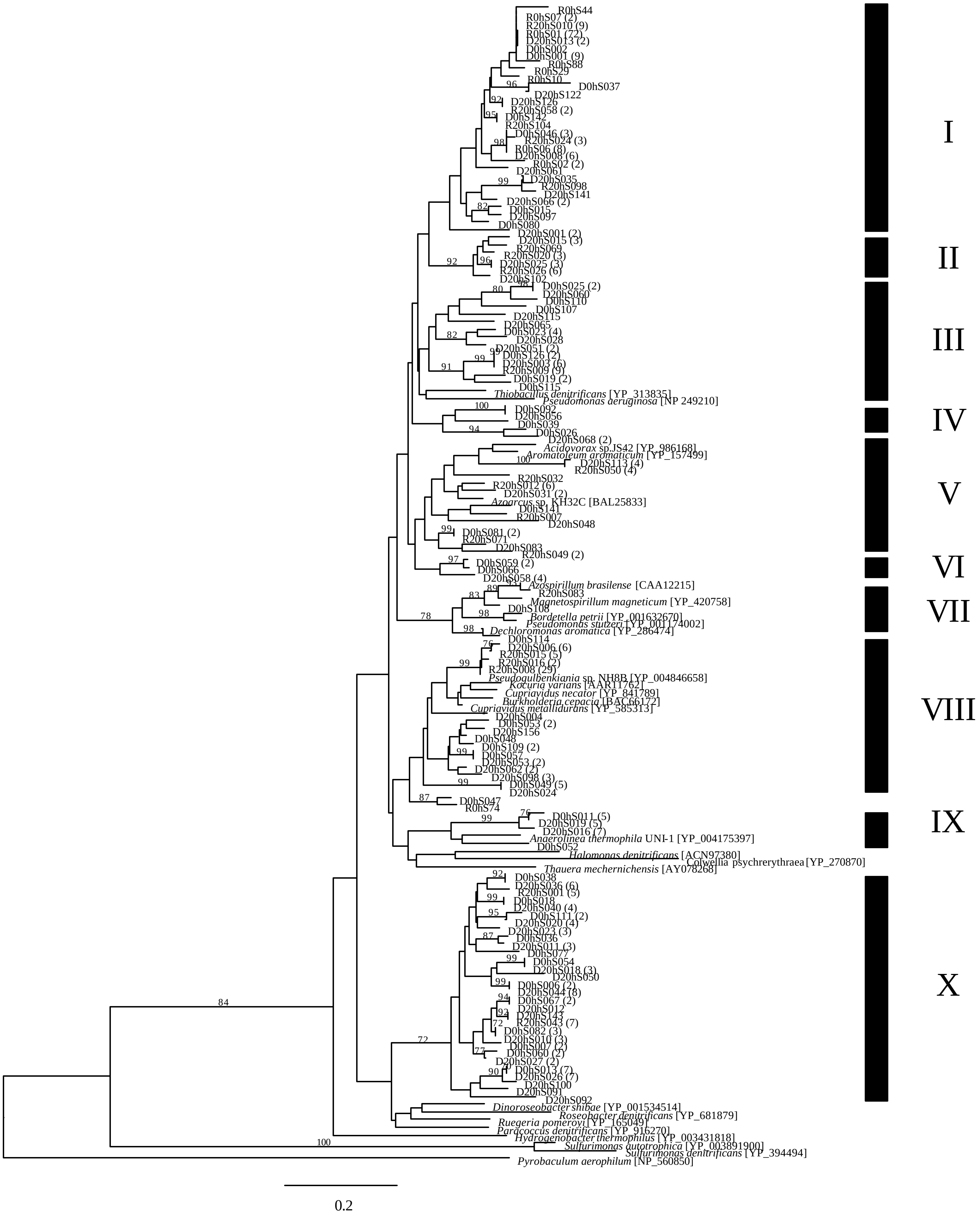

Figure S2 Neighbor-joining tree based on the deduced NirS amino acid sequences. Bootstrap values (%) were generated from 1000 replicates, and the values >70% are shown. The amino acid sequences of the outgroup member was cut off. Numbers in parenthesis represent the total number of clones in the OTU. Scale bar represents substitutions per site.

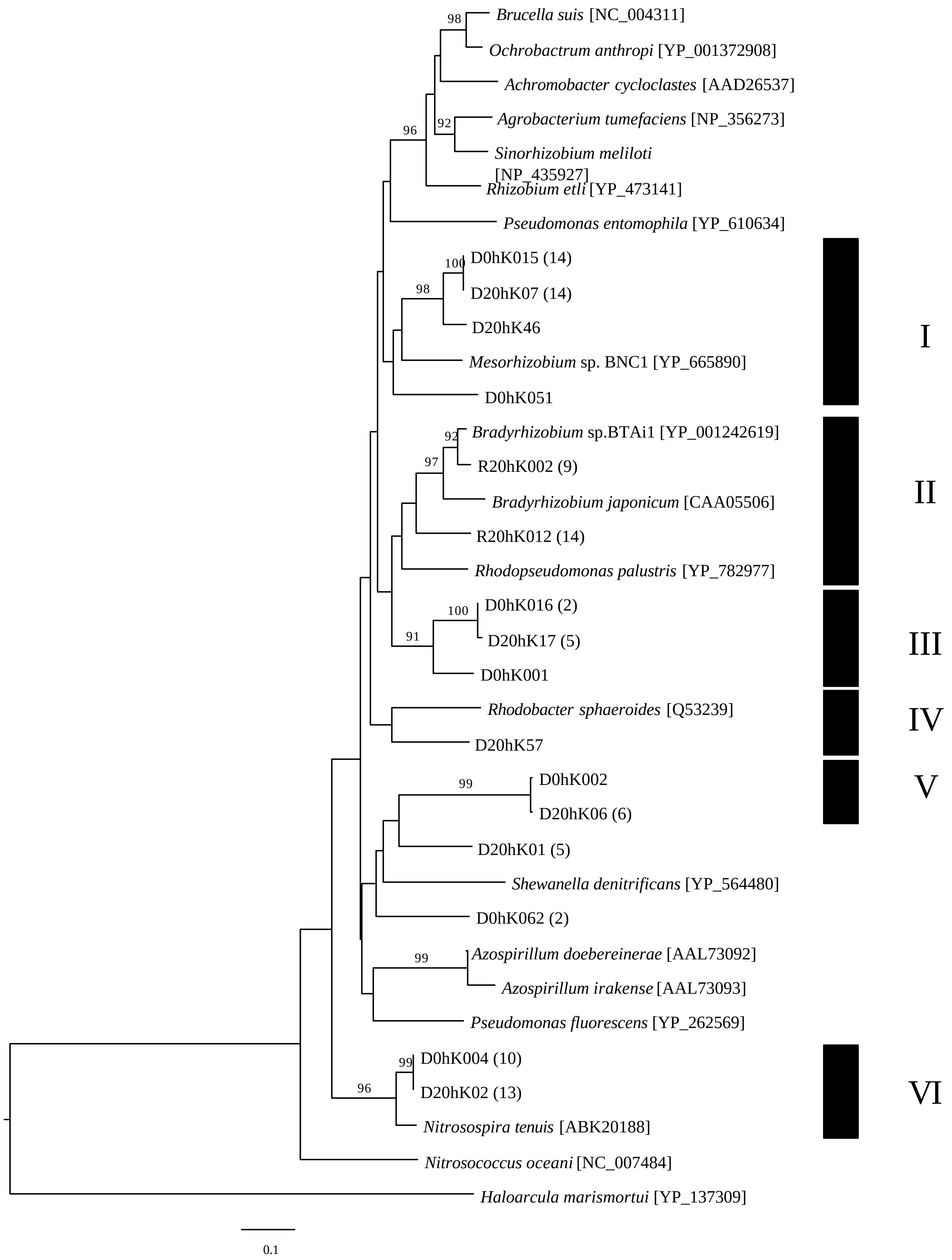

Figure S3 Neighbor-joining tree based on the deduced NirK amino acid sequences. Bootstrap values (%) were generated from 1000 replicates, and the values >70% are shown. Numbers in parenthesis represent the total number of clones in the OTU. Scale bar represents substitutions per site.

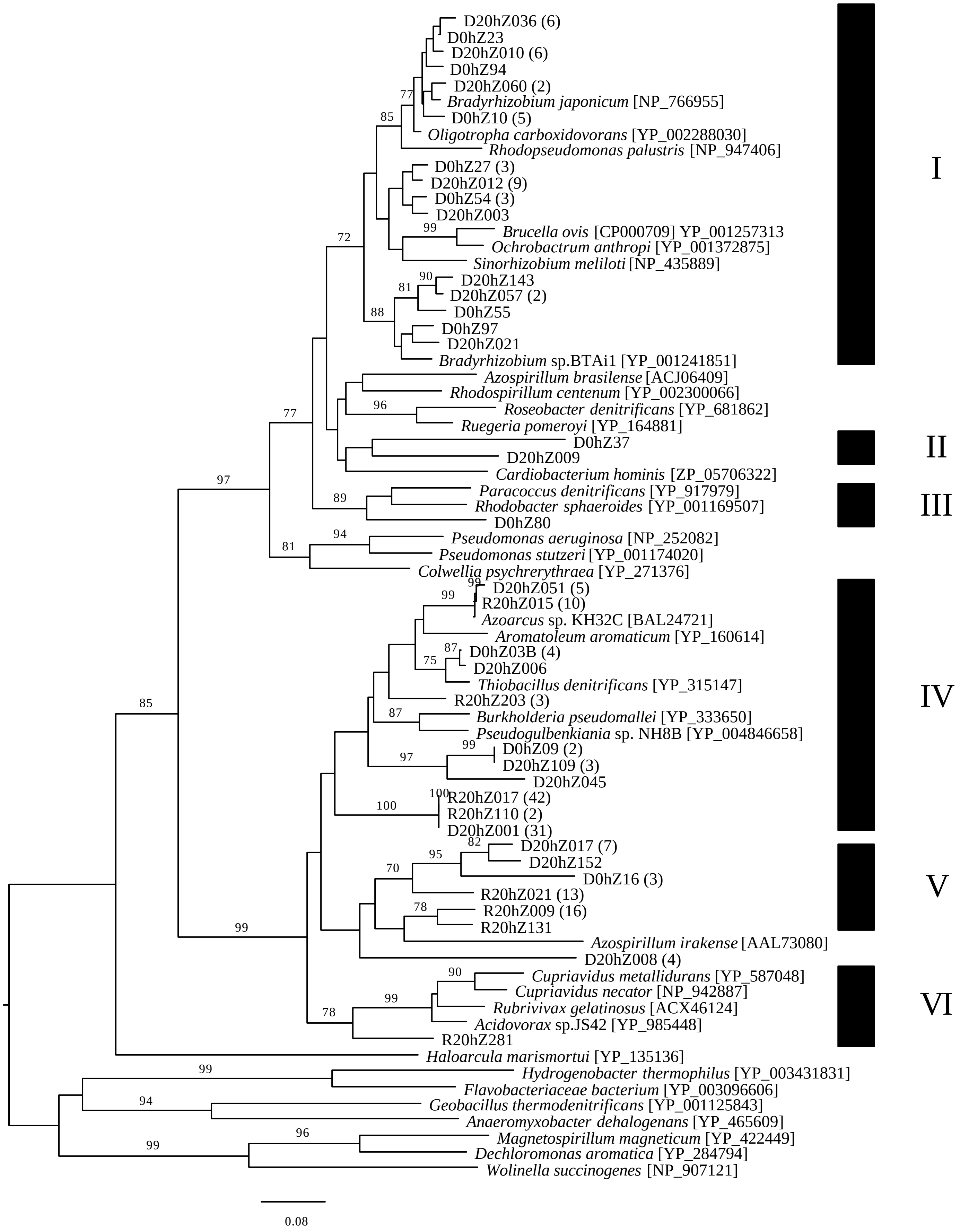

Figure S4 Neighbor-joining tree based on the deduced NosZ amino acid sequences. Bootstrap values (%) were generated from 1000 replicates, and the values >70% are shown. Numbers in parenthesis represent the total number of clones in the OTU. Scale bar represents substitutions per site.
